# Modulating Carbon Fiber Surfaces with Vinyltriethoxysilane Grafting to Enhance Interface Properties of Carbon Fiber/Norbornene–Polyimide Composites

**DOI:** 10.3390/ma17184594

**Published:** 2024-09-19

**Authors:** Jianshun Feng, Guoqiang Kong, Meng Shao, Qiubing Yu, Guang Yu, Xin Ren, Wenjie Yuan, Wenbo Liu, Xinyu Wang, Kang Wang, Dayong Li, Chengrui Di, Bo Zhu

**Affiliations:** 1 Key Laboratory for Liquid Solid Structural Evolution and Processing of Materials (Ministry of Education), School of Materials Science and Engineering, Shandong University, Jinan 250061, China; 2 Shandong Institute of Nonmetallic Materials, Jinan 250031, China

**Keywords:** composites, interfacial bonding, large-scale production, active groups, vinyltriethoxysilane

## Abstract

In this study, vinyltriethoxysilane (TEVS) was introduced onto the surface of carbon fiber using liquid-phase oxidation and impregnation methods to incorporate vinyl groups onto the carbon fiber, thereby enhancing the chemical bonding between the carbon fiber and norbornene–polyimide (PI-NA). Through a systematic study of the hydrolysis conditions and concentration of the TEVS solution, the optimal modification conditions were determined. These conditions were used to graft TEVS onto the surface of oxidized carbon fiber to prepare carbon-fiber-reinforced PI-NA composites (CF/PI-NA). The results show that when carbon fiber was treated with a 0.4 wt% TEVS solution, the interlaminar shear strength (ILSS) of the composites reached 65.12 MPa, and the interfacial shear strength (IFSS) reached 88.58 MPa, representing increases of 27.58% and 35.62%, respectively, compared to the CF/PI-NA composite materials prepared from untreated carbon fiber. It is worth noting that the modification method described in the study is simple and easy to implement, and it has the potential for large-scale continuous production applications.

## 1. Introduction

Carbon fiber/polyimide (CF/PI) composites combine the excellent properties of both carbon fiber and PI, resulting in a high-performance material with superior characteristics. Carbon fiber contributes high strength and rigidity, low density, high-temperature tolerance, outstanding fatigue performance, and good electrical and thermal conductivities [1,2,3]. PI enhances the composite with superior heat resistance, mechanical properties, exceptional electrical insulation, chemical stability, radiation resistance, and dimensional stability [4,5,6]. The synergy of these materials makes the composite highly advantageous in high-performance applications, such as aerospace, automotive, and electronics, making it an indispensable material in these industries [7].

However, the surface of carbon fiber, characterized by a non-polar, highly crystalline lamellar graphite structure, exhibits significant chemical stability, thus lacking active functional groups [8,9,10]. This characteristic leads to a lack of chemical bonding between the carbon fiber and the resin, preventing the effective transfer of external loads to the carbon fiber and resulting in the decreased mechanical performance of the composites [11,12]. By introducing active functional groups onto the surface of carbon fibers, the chemical bonding between the carbon fibers and resin can be enhanced, thereby improving the interfacial bonding strength and effectively addressing this issue [13]. In recent years, various technologies have been employed to alter the chemical composition of carbon fiber surfaces, including chemical and electrochemical oxidation, plasma treatment, gamma irradiation, and surface chemical grafting [14,15,16,17,18,19,20].

Among these methods, chemical grafting is particularly notable for its flexibility. Researchers can select specific chemicals to modify the carbon fiber’s surface, promoting chemical bonding with the reactive groups in the matrix resin. For instance, Sun et al. used an ultrasonic method to in situ polymerize polydopamine on the carbon fiber surface, introducing abundant polar groups to enhance the interfacial bonding strength between carbon fiber and polyamide 6 (PA6) [21]. Similarly, Liu et al. applied a mixed layer of polyethylenimine/polydopamine (PEI/PDA) rich in amino groups onto the carbon fiber surface via oxidative copolymerization, improving fiber–matrix interfacial interactions and wettability, thereby enhancing the interfacial performance between fiber and polyurethane (PU) [22]. Dabees et al. used a direct in situ surface modification method to graft 4-nitroaniline and 4-aminophenol onto the carbon fiber surface, enhancing the hydrogen bonding capability between carbon fiber and polyphenylene sulfide (PPS) [23]. Zhang et al. employed a one-step method to encapsulate chitosan (CS), a biomass material, on the carbon fiber surface, significantly improving the surface roughness and wettability, thus enhancing the interfacial bonding strength between carbon fiber and epoxy resin (EP) [24].

However, these methods often involve complex steps and expensive raw materials, making them unsuitable for large-scale continuous production. Silane coupling agents are a class of chemical substances capable of forming durable bonds between organic and inorganic materials. Their basic structure typically includes the following two functional groups: one that can undergo hydrolysis, such as alkoxy, halogen, or amino groups, and an organic functional group, such as vinyl or epoxy groups [25,26,27]. They are simple to use and suitable for large-scale production.

**Figure 1:**
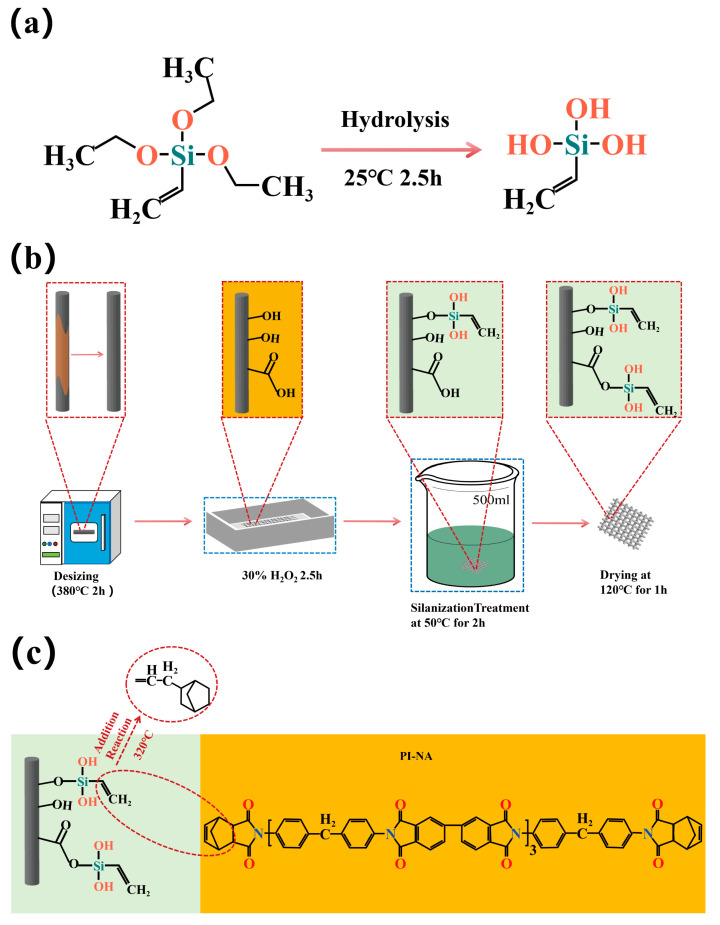
(**a**) Hydrolysis reaction of the TEVS coupling agent; (**b**) schematic of the graft of the TEVS coupling agent on the carbon fiber surface; (**c**) reaction mechanism between the carbon fiber and resin after coating with TEVS coupling agent.

In this study, a two-step method was adopted to treat carbon fiber to enhance the interfacial bonding strength between carbon fiber and PI-NA. As shown in Figure 1a,b, first, hydrogen peroxide (H_2_O_2_) solution was used for the liquid-phase oxidation of carbon fiber to increase the number of hydroxyl (-OH) and carboxyl groups (-COOH) on the carbon fiber surface thereby providing active reaction sites for the silane coupling agent. Subsequently, the carbon fiber surface was modified using a hydrolyzed solution of vinyl triethoxysilane coupling agent (TEVS) by a dipping method, which allows the vinyl group to be introduced into the carbon fiber surface by reacting with the surface active sites of the carbon fiber (-COOH and -OH) after hydrolysis of the TEVS. The vinyl group in TEVS can react with the norbornene group in PI-NA to form a bridge between the carbon fiber and PI-NA, thereby improving the interfacial bonding strength between the carbon fiber and PI-NA.

Therefore, in this paper, the hydrolysis and condensation conditions of TEVS are discussed in depth. The carbon fiber surface reaction group is provided by liquid-phase oxidation, and then TEVS is introduced into the carbon fiber surface, and CF/PI-NA composite materials are prepared. The influence of the concentration of coupling agent solution on the interface of the composite material is studied, and the interface enhancement mechanism is revealed.

## 2. Experiment and Preparation

### 2.1. Materials

Carbon fiber based on PAN (T700, 12 K, d = 7 μm) was obtained from Guangwei Expansion Fiber Co., Ltd., Weihai, China. Triethoxyvinylsilane (TEVS, 97%) and acetic acid (HOAc, AR) were supplied by Macklin Biochemical Co., Ltd., Shanghai, China. Ammonia (NH_3_.H_2_O, 28%) was purchased from Dezhou Wanyu City Chemical Co., Ltd., Dezhou, China. Hydrogen peroxide (H_2_O_2_, 30%) solution was purchased from Henan Huize Bioengineering Co., Ltd., Zhengzhou, China. Absolute alcohol (AR) was purchased from Sinopharm Chemical Reagent Co., Ltd., Shanghai, China. Norbornene polyimide (PI-NA) solution (50%) was purchased from the Institute of Chemistry Chinese Academy of Sciences, Beijing, China. Deionized water is self-made.

#### 2.1.1. Desizing of Carbon Fiber

The carbon fiber bundle was placed in a high-temperature oven, and a desizing treatment at 380 °C for 2 h was performed. The resulting carbon fiber was named De-CF.

#### 2.1.2. Preparation of TEVS Polymer Film

A mixed solution of ethanol and H_2_O (volume ratio: 4:1) was prepared, and the PH of the solution was adjusted using acetic acid and ammonia. TEVS was added dropwise to solutions of different pH values (4.5, 5.5, 7, 8, and 9) that were prepared for a 1.0 wt% TEVS solution. Subsequently, the solution was stirred and hydrolyzed at 25 °C for 2.5 h to obtain a TEVS hydrolysis solution. Finally, the hydrolysis solution was poured into a glass culture dish and dried in a vacuum oven (01-E, Honghua, China) for 2 h to obtain the TEVS polymer film.

#### 2.1.3. Surface Modification of Carbon Fiber by TEVS

The carbon fiber, after desizing, was immersed in a 30% H_2_O_2_ solution and oxidized at 60 °C for 2 h. The resulting carbon fiber was named CFO. Furthermore, the TEVS hydrolysate solution with pH = 5.5 and concentrations of 0.1 wt%, 0.4 wt%, 0.7 wt%, and 1.0 wt% were prepared at 25 °C for 2.5 h. Then, the CFO was immersed in the previously mentioned coupling agent solution at 50 °C for 2 h. Subsequently, these carbon fibers were rinsed with deionized water and placed in an oven to dry for 6 h at 50 °C, followed by an increase in temperature to 120 °C for an additional 2 h. After the oven cooled to room temperature, the modified carbon fiber was washed three times with deionized water to remove the excess silane coupling agent. The carbon fiber modified with different concentrations of the silane coupling agent were named TEVS-0.1, TEVS-0.4, TEVS-0.7, and TEVS-1.

#### 2.1.4. Carbon Fiber/PI-NA Composite Preparation

The preparation process for the CF/PI-NA composite material is illustrated in Figure S1a. Firstly, De-CF, TEVS-0.1, TEVS-0.4, TEVS-0.7, and TEVS-1 were used, with 24 beams each, and coated with PI-NA resin to prepare the prepreg. The mass ratio of resin to carbon fiber was 6:4. Then, the carbon fibers coated with resin were dried in a 45 °C oven for 12 h to remove the solvent and obtain a carbon fiber prepreg. The carbon fiber prepregs were placed in a mold, heated, pressed using a molding machine, and, finally, CF/PI-NA composite material was obtained. The molding process parameters are shown in Figure S1b. Temperatures were maintained at 120 °C, 200 °C, 280 °C, and 320 °C for 100 min, 90 min, 90 min, 60 min, and 240 min, respectively. A pressure of 5 MPa was applied once the temperature reached 280 °C. The CF/PI-NA composites were subsequently cut into 20 × 10 × 2 mm specimens, as depicted in Figure S1c. The composites made from carbon fibers treated with different concentrations of the silane coupling agent were named TEVS-0.1/PI-NA, TEVS-0.4/PI-NA, TEVS-0.7/PI-NA, and TEVS-1/PI-NA, respectively. The composite made from untreated carbon fibers was named De-CF/PI-NA.

## 3. Experimental Characterization

### 3.1. ILSS

The interlaminar shear strength (ILSS) of the composites, obtained through the short beam strength (Fsbs) test, was measured using Universal Testing Equipment (CMT4204, MTS-SANS, Shenzhen, China) according to the JC/T 773–2010 standard [28]. These specimens were placed on supports, the span was 10 mm, and a bending load was applied centrally at a loading rate of 1 mm/min, as shown in Figure S1d. The test was halted when the specimen failed or the maximum load was reached, at which point the failure mode and peak load were recorded. Each sample was tested at least 5 times, and the average value was taken. The ILSS calculation result is shown in the following formula:

Fsbs = 3P/4bh(1)

where Fsbs is the short beam strength (MPa), P is the failure load (N), b is the width of the specimen (mm), and h is the thickness of the specimen (mm).

### 3.2. IFSS

The interfacial shear strength (IFSS) of the modified carbon fiber and resin was determined by a micro-bonding test (model HM410, Tokyo, Japan). The preparation method and testing process of the IFSS sample are shown in Figure S2. The CF monofilament was fixed on an iron frame, a PI-NA/ethanol solution with a concentration of 20 w% was prepared, the solution was dipped and brushed onto the surface of the CF monofilament, and under the action of surface tension, the solution dissolved and formed droplets on the surface of the carbon fiber. The iron frame was then transferred to an oven and kept at 120 °C, 200 °C, 280 °C, and 320 °C for 1 h each to promote the conversion of polyimide acid (PAA) to PI-NA and remove the solvent. The prepared carbon fiber monofilament was removed from the iron frame, pasted on a suitable cardboard frame, and installed on the interface performance evaluation device for a drawing test (Figure S2a,b). During the test, a symmetric droplet with a length of 40–60 um was selected, and the drawing speed was 1 um/s (Figure S2c). Each sample was tested at least 5 times, and the average value was taken. The IFSS result was calculated by recording the maximum load of the resin droplet during the drawing process. The calculation formula is shown in the following formula:

IFSS = F/πdl(2)

where F is the maximum load (N) when the fiber is pulled out, d is the diameter of the fiber filament (m), and L is the length of the resin droplet (m). 

### 3.3. Single-Fiber Tensile Testing

The tensile strength of the carbon fiber was measured using a Fiber Tensiometer (XQ-1C, XinXian, China). The stretching standard for carbon fiber monofilament is GB/T 31290-2022 [29]. The procedure for preparing a single-carbon-fiber tensile sample is illustrated in Figure S3. A single fiber was carefully extracted from the CF fabric, and both ends were affixed to a piece of test paper. The test span was set at 20 mm. This setup was then secured to the grips of the fiber tensiometer, operating at a tensile rate of 2 mm/min. For each sample, at least 35 individual fibers were tested, and the average tensile strength was calculated. The strength was determined using the following equation:

(3) $$\sigma =4F/\pi d^{2}$$

where σ is the single CF’s tensile strength (Pa), F is the maximum breaking load (N), and d is the single CF’s diameter (m). The results were statistically analyzed using the Weibull distribution model.

### 3.4. FTIR of TEVS Polymer Film

The hydrolysis and condensation polymerization mechanisms of the TEVS coupling agent were investigated using Fourier Transform Infrared Spectroscopy (FTIR, VRRTEX-70, Bruker, Baltimore, MD, USA). FTIR was conducted using the KBr pellet method, scanning the wavenumber range from 400 to 4000 cm^−1^.

### 3.5. Hydrolytic Conductivity of Hydrolysis Solutions of TEVS

The changing trend in the coupling agent’s hydrolytic conductivity was determined using a Push-Button Bench Conductivity Meter (LC-EC-2S, Shengke, Shanghai, China). Hydrolysis solutions of TEVS with a concentration of 1% and a pH values of 4.5, 5.5, 7, 8, and 9 were prepared, and the pH value was adjusted using ammonia water and acetic acid. The Push-Button Bench Conductivity Meter (LC-EC-2S, Shengke, Shanghai, China) was used to record changes in conductivity of the hydrolysis solutions of TEVS silane coupling agent at different pH values mentioned above, recording data every 10 min at 25 °C.

### 3.6. SEM Testing

The surface morphology of the carbon fiber and the fracture surface morphology of the composites were examined using a Field Emission Scanning Electron Microscope (FE-SEM, model SU-70, JEOL, Ibaraki, Japan). The SEM testing used a 15 KV test voltage, and the sample was pretreated with gold spray for 30 s before testing.

### 3.7. XPS Testing

X-ray photoelectron spectroscopy (XPS, AXIS ULTRA, HORIBA Jobin Yvon, Paris, France) was utilized to investigate the elemental and functional group changes on the carbon fiber surface. The X-ray emission was performed using a Mg Kα line (12 kV, 200 W) anode from a non-monochromatic ultra-high-vacuum (UHV) source. After an initial survey scan, high-resolution scans were conducted at a pass energy of 10 eV to characterize the chemical states of the sample surfaces, including chemical composition and element valence states. The excitation energy used was 1253.6 eV. To prevent charging during analysis, the fiber-shaped samples were cut to appropriate sizes and mounted on sample holders using copper tape.

### 3.8. Water Contact Angle

The processes for preparing samples and measuring the water contact angle is illustrated in Figure S4. The carbon fiber tow was laid out flat and adhered side by side onto glass slides. During this process, a certain amount of tension was applied to the carbon fiber bundles to ensure that the top surface remained smooth and level. The carbon fiber tows should be closely packed with no gaps between them. The prepared samples for the water contact angle measurement were placed horizontally on the stage of the Contact Angle Meter (JC2000D1, Kezhong, Shanghai, China) for testing. When the water droplet stabilizes on the carbon fiber tow for 0.1 s, the photo taken serves as the result. The water drop is about 10 μL. The contact angle was determined by performing a five-point fitting on the contact angle images using the ellipse method. Figure S5 shows the workflow of this work.

## 4. Results

As shown in Figure 1a, the TEVS coupling agent hydrolysis could form reactive silanol groups (Si-OH). As shown in Figure 1b, after desizing, the carbon fibers were treated with a 30% H_2_O_2_ solution, resulting in the generation of abundant -COOH and -OH on the surface, providing active sites for subsequent chemical reactions. Subsequently, the carbon fibers were treated with the hydrolyzed solution of TEVS coupling agent, where the Si-OH generated from the hydrolysis of TEVS reacted with the -OH on the carbon fiber surface through condensation reactions to form ether bonds [30,31]. During the subsequent heating process, the TEVS coupling agent adsorbed on the carbon fiber surface further can reacted with the -COOH on the carbon fiber, forming stable covalent bonds through esterification. As illustrated in Figure 1c, during the curing process of the PI-NA resin, the vinyl groups in the TEVS generated free radicals at high temperatures, which then reacted with the norbornene groups at the ends of the PI-NA molecular chains through radical addition, forming new covalent bonds. This series of treatment steps, by introducing active functional groups on the carbon fiber surface and leveraging the chemical reactions between TEVS and PI-NA, construct stable chemical bridges [32], significantly enhancing the overall performance of the CF/PI-NA composites, including improved interfacial bonding strength and increased mechanical properties and stability of the material.

**Figure 2:**
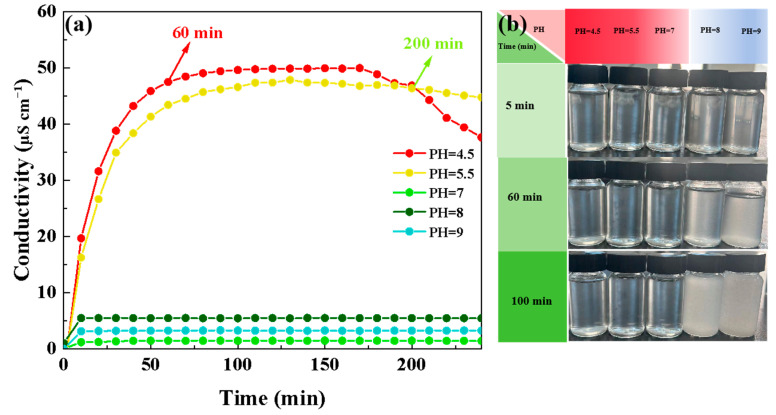
(**a**) Electrical conductivity of the TEVS hydrolysis solution at different pH values; (**b**) photographs of the TEVS hydrolysis solution at different stages of hydrolysis.

The conductivity test on the silane-coupling-agent hydrolyzed solution was used to analyze the optimum hydrolysis and polycondensation parameters of TEVS. As shown in Figure 2a, the conductivity of the TEVS hydrolysates under both acidic and basic conditions was higher than that under neutral conditions, indicating that both acidic and basic environments are conducive to the hydrolysis of TEVS. Under acidic conditions, the conductivity of the hydrolyzed solution reached stability after 60 min. In acidic conditions, the silicon atom in the TEVS first needs to be protonated, forming a positively charged silicon–oxygen intermediate. This intermediate makes water molecules more nucleophilic to attack silicon atoms and promotes the hydrolysis of TEVS to produce rich Si-OH, thus improving the conductivity of the hydrolyzed solution of TEVS [33]. In addition, at PH = 4.5, the conductivity of the TEVS hydrolyzed solution decreased after 200 min, which may be due to the production of a large number of Si-OH, resulting in TEVS polycondensation. However, under alkaline conditions, it is easy to lead to large-scale polycondensation of TEVS, so that Si-OH is consumed, resulting in the low conductivity of the hydrolyzed solution. As shown in Figure 2b, under alkaline conditions, after 5 min of hydrolysis, the hydrolyzed solution of TEVS became cloudy. Moreover, with the increase in the time to 100 min, the turbidity phenomenon was aggravated, which is attributed to the fact that alkaline easily causes the large-scale condensation of TEVS and, thus, become turbid. On the contrary, under acidic conditions, as the time increased from 5 min to 100 min, the solution became more homogeneous and thorough.

**Figure 3:**
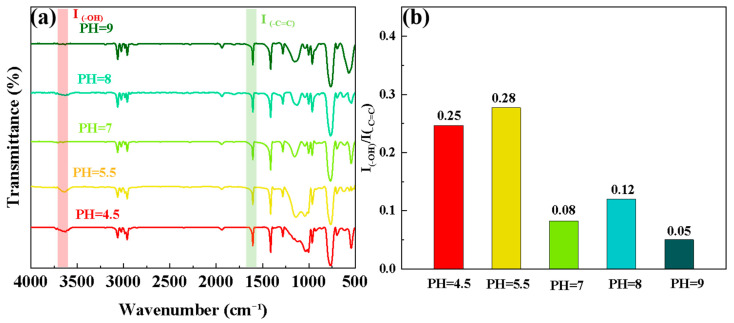
(**a**) FTIR spectra of TEVS polymer film at various pH values; (**b**) intensity ratio of -OH and -C=C absorption peaks (I_(-OH)_/I_(-C=C)_) named R in the FTIR spectra.

In order to further analyze the mechanism of the above phenomenon, the hydrolysates at different PH values were dehydrated and underwent polycondensation to form TEVS polymer films, which were tested by FTIR. Figure 3a shows the FTIR spectra of the TEVS polymer films under different pH conditions. The polymer films do not exhibit the -CH3 absorption peak, and a new -OH absorption peak appears at 3649 cm^−1^, while the positions of the other absorption peaks remain almost unchanged. This indicates that TEVS undergoes hydrolysis and condensation reactions under acidic, neutral, and alkaline conditions. Additionally, variations in the intensity of the -OH absorption peak in hydrolysis solutions with different pH values were observed. To further analyze this phenomenon, the -C=C absorption peak at 1606 cm^−1^ was selected as a reference, and the ratio of the -OH absorption peak to the -C=C absorption peak intensity was calculated [34]. As shown in Figure 3b, The R values at pH = 4.5 and pH = 5.5 were 0.25 and 0.28, respectively, which are higher than those at pH = 7 (R = 0.08), pH = 8 (R = 0.12), and pH = 9 (R = 0.05), which indicates that the -OH content in the TEVS films was significantly higher in acidic environments than in neutral and alkaline environments. This is mainly because, in acidic conditions, a large number of H^+^ make the silicon atoms in the TEVS more easily protonated, which then get attacked by oxygen atoms from water molecules, converting ethoxy groups into -OH. The higher the acidity, the more pronounced this conversion, thus increasing the -OH content. However, at excessively high pH, silane coupling agents can undergo condensation, resulting in a decrease in -OH content, which explains why the R value of pH = 4.5 was lower than that of pH = 5.5. On the other hand, in alkaline environments, although OH^−^ ions can promote the hydrolysis of TEVS, high concentrations of OH- ions interfere with the stability of the hydrolysis products. This not only hinders the forward movement of the hydrolysis equilibrium but also accelerates the condensation reaction, resulting in precipitation. Therefore, as the PH value increases, the hydrolysis products of the TEVS gradually decrease, and the -OH content correspondingly decreases.

**Figure 4:**
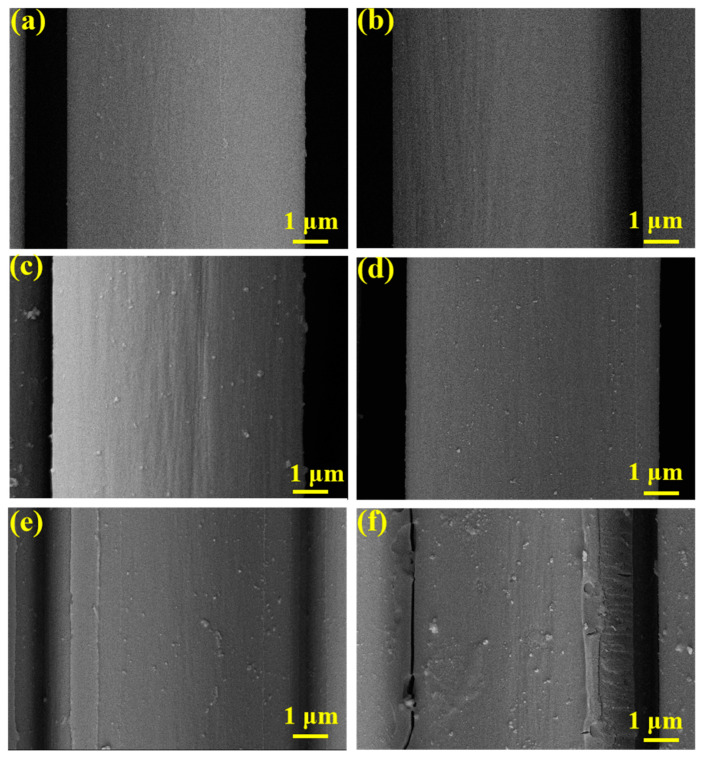
SEM images: (**a**) De-CF; (**b**) CFO and carbon fiber coated by TEVS of (**c**) 0.1%, (**d**) 0.4%, (**e**) 0.7%, and (**f**) 1%.

The SEM was used to analyze the load of the TEVS on the carbon fiber surface. As shown in Figure 4a,b, the carbon fiber still had residual carbon deposits after the high-temperature desizing, but these residues were completely removed after treatment with hydrogen peroxide solution, revealing distinct grooves on the surface. As depicted in Figure 4c, when treated with a 0.1% TEVS silane coupling agent solution, the carbon fiber surface does not exhibit a noticeable coating but instead forms aggregated particles. This may be due to the ease of hydrolysis and condensation reactions at low concentrations of the silane coupling agent, leading to particle formation. As shown in Figure 4d, after treatment with a 0.4% TEVS solution, a uniform coating forms on the carbon fiber surface, making the grooves shallower. This indicates that higher concentrations of TEVS help in forming a more complete coating. Figure 4e,f illustrate that with further increases in the concentration of the silane coupling agent, the carbon fiber surface was covered with a thicker TEVS layer, and the fibers exhibited adhesion between them. This thick coating and adhesion can affect the infiltration and interfacial bonding of the PI-NA resin, potentially leading to a decrease in the mechanical properties of the composite material [35].

**Figure 5:**
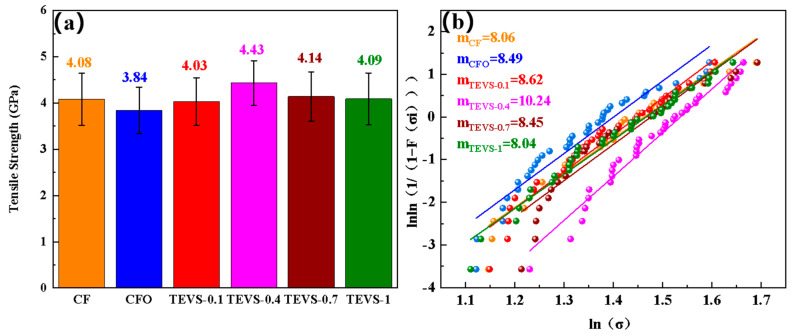
(**a**) Monofilament tensile strength and (**b**) Weibull distribution fitting results for the carbon fiber.

To verify the effect of the surface treatment on the strength of the carbon fiber, tensile strength tests were conducted on a single carbon fiber before and after modification, as shown in Figure 5a. The results indicate that the strength of the carbon fiber significantly decreased after the surface oxidation treatment. This is because surface oxidation etches the carbon fiber, increasing surface defects, leading to uneven stress distribution during tensile loading, and thereby reducing strength. However, after the TEVS modification, the strength of the carbon fiber initially increased and then decreased. A low concentration (0.4%) of TEVS coating can repair surface defects on carbon fiber, reducing the stress concentration during loading. Conversely, an excessively high concentration of the coupling agent forms a thick and uneven brittle coating on the surface of the carbon fiber, causing stress concentration and leading to fiber fracture. The Weibull fitting distribution was used to evaluate the dispersion of carbon fiber monofilament strength, as shown in Figure 5b. The higher the m_f_ value, the lower the dispersion of monofilament strength. When the concentration of the silane coupling agent was 0.4%, the dispersion was the smallest (m_TEVS-0.4_ = 10.24). At silane coupling agent concentrations of 0.1%, 0.7%, and 1%, the m_f_ values were 8.62, 8.45, and 8.04, respectively. This indicates that both excessively high or low concentrations of the silane coupling agent cannot effectively improve the dispersion of the carbon fiber’s monofilament strength [36].

**Figure 6:**
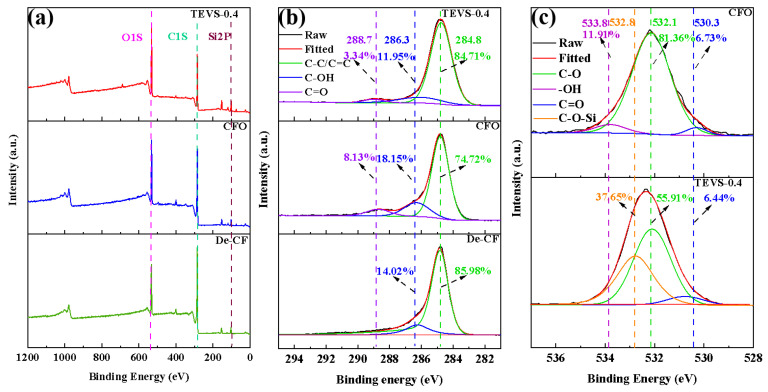
(**a**) Wide-scan XPS spectra; (**b**) C1s peak spectra of De-CF, CFO, and TEVS-0.4; (**c**) O1s peak spectra of CFO and TEVS-0.4.

The XPS spectra in Figure 6a reveal the relative elemental composition of different CF samples, with all fibers containing C, O, N, and Si. According to Table 1, the O/C ratios for CFO and TEVS-0.4 were 0.31 and 0.51, respectively, both higher than the O/C ratio of De-CF (0.23). These ratios indicate that the liquid-phase oxidation treatment increased the number of polar groups on the CF surface and that TEVS grafting was successful. To investigate the impacts of the treatment methods on the surface chemical composition of the CF, the modified fibers were analyzed using XPS for their C spectra. As shown in Figure 6b, the peaks for C-C/C=C in the aromatic rings, C-OH groups, and C=O groups appeared at 284.8 eV, 286.3 eV, and 288.7 eV, respectively [37,38]. In terms of the functional group composition, the De-CF surface exhibits a high proportion of C-C bonds, with only 14.02% C-OH detected. After the oxidation treatment, the C content on the carbon fiber surface decreased, while the levels of C-OH and C=O increased. This indicates that the liquid-phase oxidation treatment can convert some of the carbon structures on the carbon fiber surface into -OH and -COOH, providing the necessary chemical active sites for subsequent reactions. Additionally, with the introduction of the TEVS coupling agent, the C-OH peak on the carbon fiber surface diminished (11.04%). This can be attributed to the esterification reaction between the Si-OH groups in the silane coupling agent and the -COOH on the carbon fiber surface. To further verify whether TEVS was grafted onto the carbon fiber surface, the O elements were further analyzed. As shown in Figure 6c, the -OH content on the surface of the carbon fiber after the liquid-phase oxidation treatment was 11.91%. However, for the carbon fiber treated with TEVS, the -OH peak disappeared, and a C-O-Si peak appeared at 532.8 eV. This indicates that TEVS was successfully grafted onto the carbon fiber surface [39].

**Figure 7:**
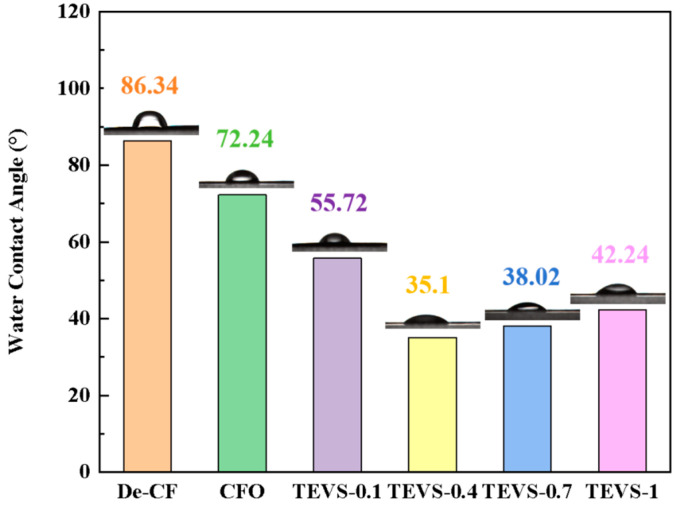
Carbon fiber surface water contact angle after 0.1 s.

By measuring the water contact angles of the carbon fiber before and after treatment, the effect of the TEVS grafting on the surface wettability of the carbon fiber can be evaluated. As shown in Figure 7, the desized carbon fiber exhibited high inertness, with a water contact angle of 86.34°. After the treatment with H_2_O_2_ solution, the water contact angle of the carbon fiber surface decreased to 72.74°. This is because the liquid-phase oxidation treatment introduced abundant oxygen-containing groups on the carbon fiber surface, increasing its polarity and, thus, improving its wettability. With the increasing content of silane coupling agent grafted onto the carbon fiber, the contact angle initially decreased and then increased. When treated with a 0.4% TEVS concentration, the contact angle reached the lowest value of 35.1°. This trend is attributed to the fact that the grafting of the silane coupling agent increased the number of polar groups on the carbon fiber surface [40]. However, an excessive amount of silane coupling agent filled the grooves on the carbon fiber surface, reducing its surface roughness. As a result, the contact angle first decreased and then increased.

**Figure 8:**
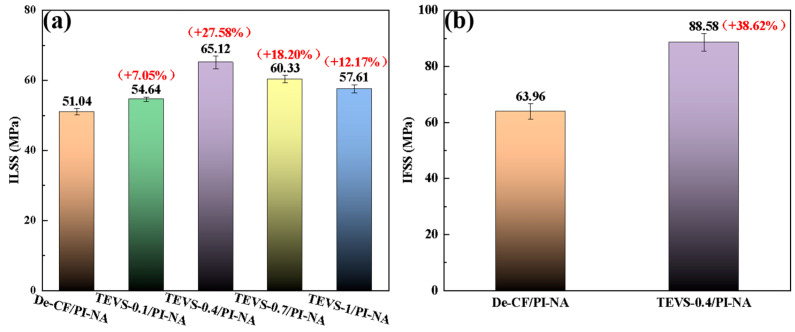
(**a**) The ILSS of the CF/PI-NA composites; (**b**) IFSS strength of the De-CF/PI-NA and TEVS-0.4/PI-NA.

The ability of CF/PI-NA to bear stress depends on the bonding strength between the fibers and the resin. The ILSS test was used to evaluate the interfacial bonding strength between the carbon fiber and PI-NA resin before and after modification. As shown in Figure 8a, the ILSS value of the De-CF/PI-NA composites was only 51.04 MPa because of the inert surface of the desized carbon fiber, which lacks effective polar groups to connect the carbon fiber and PI-NA. With the increase in the amount of TEVS grafted on the carbon fiber surface, the ILSS value of the composites increased. The TEVS-0.4/PI-NA composites exhibited the best ILSS performances, with an ILSS value of 65.12 MPa, which is 27.58% higher than that of the De-CF/PI-NA composites. However, with further increases in the TEVS load on the carbon fiber surface, the ILSS values of the TEVS-0.7/PI-NA and TEVS-1/PI-NA composites decreased to 60.33 MPa and 57.61 MPa, respectively. These results can be attributed to the excessive silane coupling agent coating, which leads to the bonding of carbon fiber bundles and affects the contact between the resin and carbon fiber. Additionally, the excess coupling agent caused brittle interfacial fractures between the carbon fiber and resin under stress, leading to the degradation of the interfacial properties of the CF/PI-NA composites. The IFSS test was further used to evaluate the interfacial bonding strength between the modified carbon fiber and the resin. As shown in Figure 8b, the IFSS value of De-CF/PI-NA was 63.96 MPa, while that of TEVS-0.4/PI-NA was 88.58 MPa, representing an increase of 38.62% compared to De-CF/PI-NA. This improvement is attributed to the effective chemical bonding formed between the carbon fiber and PI-NA, which facilitated the transfer of stress from the resin to the carbon fiber.

**Figure 9:**
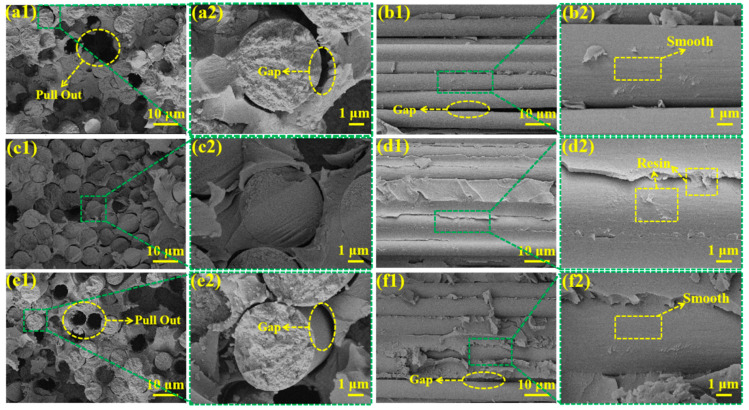
The SEM graphics of the fracture surface in the radial direction: (**a1**,**a2**) De-CF/PI-NA composites, (**c1**,**c2**) TEVS-0.4/PI-NA, and (**e1**,**e2**) TEVS-1/PI-NA; in the weft direction: (**b1**,**b2**) De-CF/PI-NA, (**d1**,**d2**) TEVS-0.4/PI-NA, and (**f1**,**f2**) TEVS-1/PI-NA.

SEM was used to analyze the radial and transverse fracture surface morphologies of the composites to investigate the failure and fracture behavior of the CF/PI-NA composites under stress. As shown in Figure 9a1,a2, on the radial fracture surface of the De-CF/PI-NA composite, numerous holes resulting from fiber pull-out can be observed, with significant gaps between the fibers and the resin. Similarly, as shown in Figure 9b1,b2, the transverse fracture surface displays smooth fiber surfaces with only a small amount of residual resin. These results indicate that the surface of the desized carbon fiber lacked active groups, resulting in a low interfacial bonding strength with PI-NA, which leads the formation of a weak interface between the carbon fiber and PI-NA, thereby reducing the interlaminar ILSS and IFSS of the De-CF/PI-NA. In contrast, as shown in Figure 9c1,c2, the radial fracture surface of the TEVS-0.4/PI-NA exhibited tight bonding between the carbon fiber and the resin, with a smooth fracture surface and no significant fiber pull-out. The corresponding transverse fracture surface, shown in Figure 9d1,d2, reveals a substantial amount of residual resin on the carbon fiber. This indicates that the strong and stable interface formed between the carbon fiber and PI-NA resin was due to robust chemical bonding, which effectively transfers stress from the resin to the carbon fiber and mitigates the propagation of interfacial cracks, resulting in a large amount of resin residue on the surface of the carbon fibers. As shown in Figure 9e1,e2, on the radial fracture surface of the TEVS-1/PI-NA composite material, a small number of pores can also be observed because of fiber pull-out, and there are obvious gaps between the fibers and the resin. Similarly, as shown in Figure 9f1,f2, the transverse fracture surface shows less residual resin on the fiber surface, which can be attributed to the formation of an excessively thick coupling agent coating on the carbon fiber surface, which increases the brittleness of the carbon fiber and PI-NA resin interface and reduces the bonding strength between the carbon fiber and the resin.

## 5. Conclusions

This study proposes a novel method for modifying the surface of carbon fiber by grafting vinyl groups onto it using liquid-phase oxidation and coupling agent methods, thereby enhancing the interfacial properties of CF/PI-NA composites. H_2_O_2_ solution is used for liquid-phase oxidation of carbon fibers, thereby endowing the surface of carbon fibers with abundant reactive groups (-OH, -COOH), providing numerous reaction sites for TEVS chemical grafting. Grafting TEVS onto the carbon fiber surface not only repairs surface defects and enhances the tensile strength of carbon fiber but also increases the number of vinyl groups on the carbon fiber surface, which can undergo an additional reaction with the norbornene group in PI-NA at high temperatures, thereby strengthening the chemical bonding between the carbon fiber and PI-NA. In addition, the optimal hydrolysis conditions for TEVS were explored, and it was proven that a pH value of 5.5 is beneficial for TEVS hydrolysis to produce more abundant Si-OH, thereby introducing more effective chemical reaction groups when TEVS is loaded onto the surface of carbon fibers. Treating carbon fiber with a 0.4 wt% TEVS solution significantly improved the surface properties of the carbon fiber, resulting in the ILSS and interfacial shear strength (IFSS) of the CF/PI-NA composites increasing by 27.58% and 35.62%, respectively, compared to De-CF/PI-NA. These improvements in the interfacial and interlaminar shear properties are attributed to the enhanced interfacial bonding between the carbon fiber and PI-NA, forming a robust stress transfer bridge between the carbon fiber and the resin, thereby inhibiting crack propagation at the interface. This method is simple and easy to implement, with the potential for large-scale continuous production applications.

## Figures and Tables

**Table 1:** Analysis of surface elements and proportions of carbon fibers.

Samples	Element Proportion (%)				O/C
	C1S	O1S	N1S	Si2P	
De-CF	73.33	16.56	5.05	5.07	0.23
CFO	67.98	21.23	5.59	5.20	0.31
TEVS-0.4	56.76	28.93	2.39	11.92	0.51

## Data Availability

The raw data supporting the conclusions of this article will be made available by the authors on request.

## Supplementary Materials

The following supporting information can be downloaded at: https://www.mdpi.com/article/10.3390/ma17184594/s1. Figure S1. (a) Preparation schematic of CF/PI-NA composite laminates (b) Temperature rise curves for the preparation of composite materials. (c) ILSS samples and (d) ILSS test diagram. Figure S2. (a) Picture of IFSS samples (b,c) Process picture of IFSS test. Figure S3. Schematic of single-CF fiber tensile sample preparation. Figure S4. Sample preparation and test method for water contact angle. Figure S5. Experimental flowchart.
