# Supplementary material for: Modulating Carbon Fiber Surfaces with Vinyltriethoxysilane Grafting to Enhance Interface Properties of Carbon Fiber/Norbornene–Polyimide Composites
Source: Materials (Basel). 2024 Sep 19;17(18):4594. doi: 10.3390/ma17184594 (PMC11433078; doi:10.3390/ma17184594)
Supplement: Supplementary file 1 [file materials-17-04594-s001.zip › materials-3145804-SI.pdf]

Modulating Carbon Fiber Surfaces with Vinyltriethoxysilane Grafting to Enhance Interface Properties of Carbon Fiber/ Norbornene–Polyimide Composites

Jianshun Feng [1], Guoqiang Kong [2], Meng Shao [2], Qiubing Yu [2], Guang Yu [2], Xin Ren [2], Wenjie Yuan [2], Wenbo Liu [2], Xinyu Wang [2], Kang Wang [2], Dayong Li [2], Chengrui Di [1] and Bo Zhu [1,*]

1 Key Laboratory for Liquid Solid Structural Evolution and Processing of Materials (Ministry of Education), School of Materials Science and Engineering, Shandong University, Jinan 250061, China

2 Shandong Institute of Nonmetallic Materials, Jinan 250031, China

* Correspondence: zhubo@sdu.edu.cn

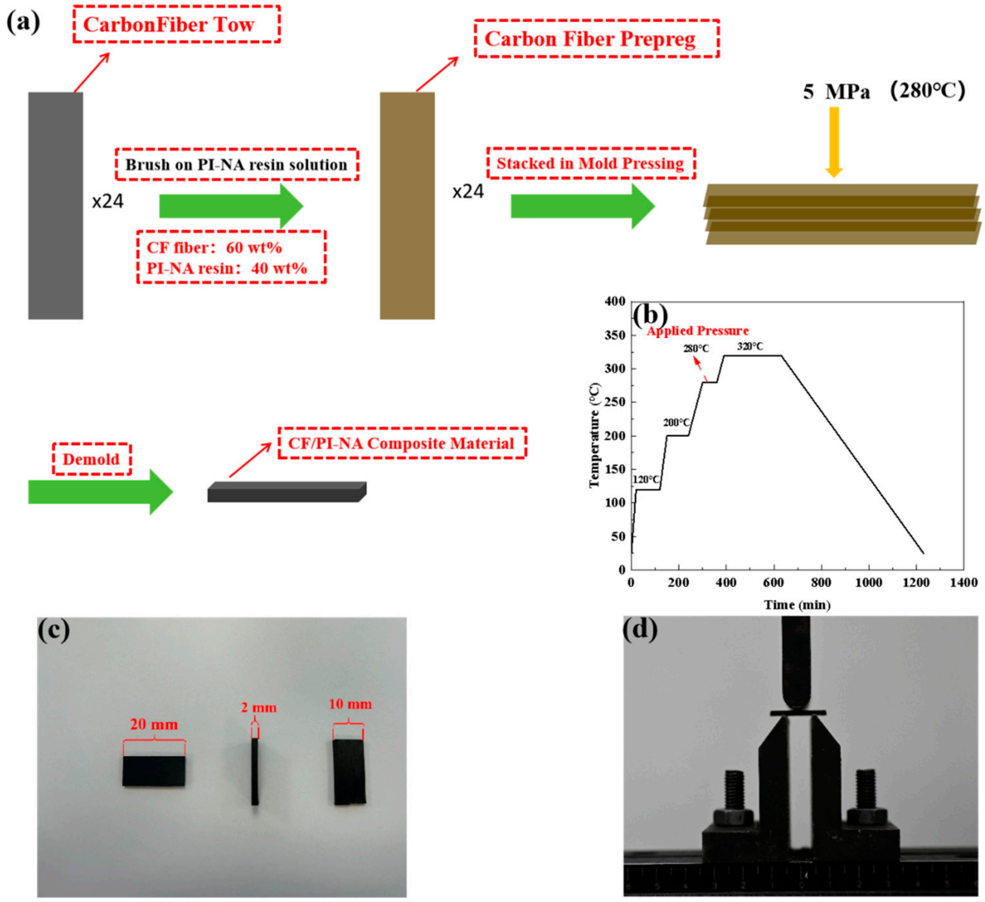

Figure S1. (a) Preparation schematic of CF/PI-NA composite laminates (b) Temperature rise curves for the preparation of composite materials (c) ILSS samples and (d) ILSS test diagram.

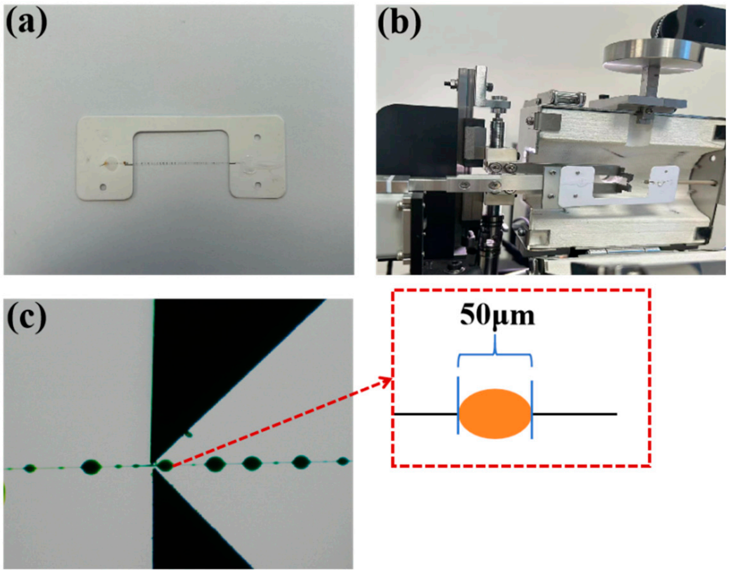

Figure S2. (a) Picture of IFSS samples (b,c) Process picture of IFSS test.

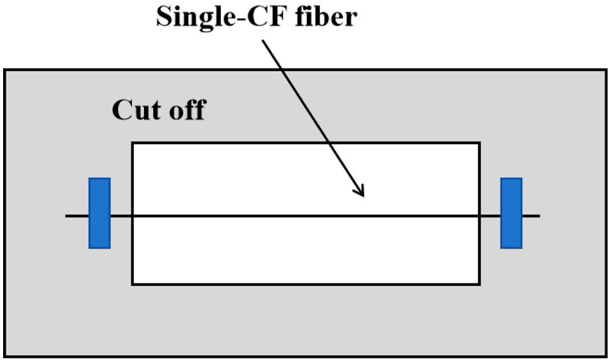

Figure S3. Schematic of single-CF fiber tensile sample preparation

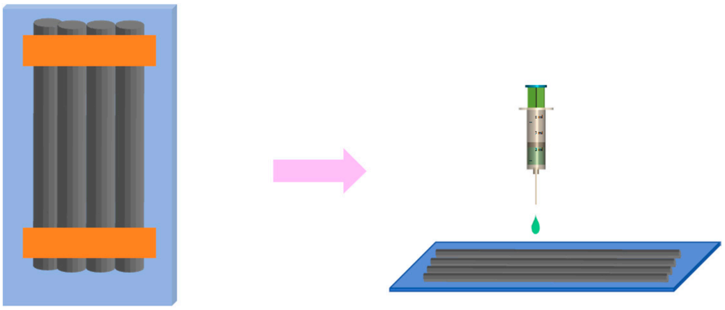

Figure S4. Sample preparation and test method for water contact angle

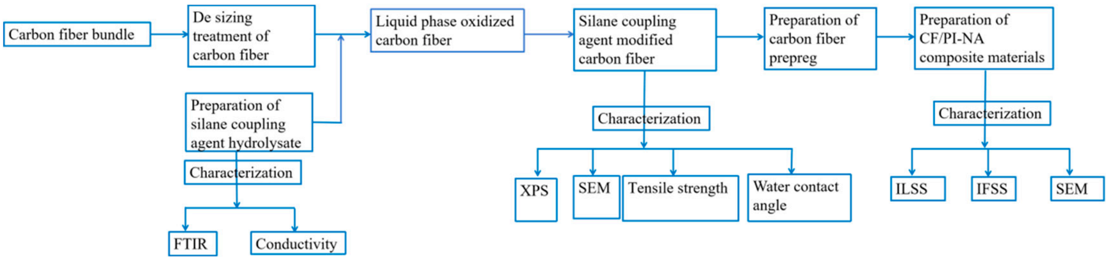

Figure S5. Experimental flowchart.
